# Mitochondrial biogenesis is required for the anchorage-independent survival and propagation of stem-like cancer cells

**DOI:** 10.18632/oncotarget.4401

**Published:** 2015-06-09

**Authors:** Arianna De Luca, Marco Fiorillo, Maria Peiris-Pagès, Bela Ozsvari, Duncan L. Smith, Rosa Sanchez-Alvarez, Ubaldo E. Martinez-Outschoorn, Anna Rita Cappello, Vincenzo Pezzi, Michael P. Lisanti, Federica Sotgia

**Affiliations:** ^1^ The Breakthrough Breast Cancer Research Unit, Institute of Cancer Sciences, University of Manchester, UK; ^2^ The Manchester Centre for Cellular Metabolism (MCCM), Institute of Cancer Sciences, University of Manchester, UK; ^3^ Department of Pharmacy, Health and Nutritional Sciences, University of Calabria, Arcavacata di Rende (CS), Italy; ^4^ The Cancer Research UK Manchester Institute, University of Manchester, UK; ^5^ The Sidney Kimmel Cancer Center, Philadelphia, PA, USA

**Keywords:** XCT790, doxycycline, drug repurposing, ERR-α, PGC1-α/β

## Abstract

Here, we show that new mitochondrial biogenesis is required for the anchorage independent survival and propagation of cancer stem-like cells (CSCs). More specifically, we used the drug XCT790 as an investigational tool, as it functions as a specific inhibitor of the ERRα-PGC1 signaling pathway, which governs mitochondrial biogenesis. Interestingly, our results directly demonstrate that XCT790 efficiently blocks both the survival and propagation of tumor initiating stem-like cells (TICs), using the MCF7 cell line as a model system. Mechanistically, we show that XCT790 suppresses the activity of several independent signaling pathways that are normally required for the survival of CSCs, such as Sonic hedgehog, TGFβ-SMAD, STAT3, and Wnt signaling. We also show that XCT790 markedly reduces oxidative mitochondrial metabolism (OXPHOS) and that XCT790-mediated inhibition of CSC propagation can be prevented or reversed by Acetyl-L-Carnitine (ALCAR), a mitochondrial fuel. Consistent with our findings, over-expression of ERRα significantly enhances the efficiency of mammosphere formation, which can be blocked by treatment with mitochondrial inhibitors. Similarly, mammosphere formation augmented by FOXM1, a downstream target of Wnt/β-catenin signaling, can also be blocked by treatment with three different classes of mitochondrial inhibitors (XCT790, oligomycin A, or doxycycline). In this context, our unbiased proteomics analysis reveals that FOXM1 drives the expression of >90 protein targets associated with mitochondrial biogenesis, glycolysis, the EMT and protein synthesis in MCF7 cells, processes which are characteristic of an anabolic CSC phenotype. Finally, doxycycline is an FDA-approved antibiotic, which is very well-tolerated in patients. As such, doxycycline could be re-purposed clinically as a ‘safe’ mitochondrial inhibitor, to target FOXM1 and mitochondrial biogenesis in CSCs, to prevent tumor recurrence and distant metastasis, thereby avoiding patient relapse.

## INTRODUCTION

Tumor-initiating stem-like cells (TICs) are a small sub-population of tumor cells that are resistant to most anti-cancer therapies, including radio- and chemotherapy, and are able to expand and regenerate tumors, after conventional therapy is completed [1-4]. As such, TICs are responsible for tumor recurrence, metastatic dissemination, and, ultimately, patient death. As a consequence, TICs have become very attractive targets for novel cancer therapies. TICs share some features with stem cells, and many studies have investigated the signaling pathways regulating their proliferation, asymmetric cell division and migrating properties, as well as their ability to undergo proliferation under anchorage-independent conditions [5-7]. In fact, this latter property is being widely exploited to isolate TICs, which are able to survive and clonally expand as tumor-spheres, when placed in non-adherent settings [8]. Tumor-spheres generated from breast cancer cells are known as mammospheres.

The metabolic requirements of TICs remain a fairly unexplored area of investigation. We and others have recently shown that TICs rely mainly on mitochondrial metabolism, as compared to the more differentiated tumor cell population [9-12]. Also, it was previously shown that the radio-resistance of TICs from gliomas [13] and breast cancers [14] correlates with higher mitochondrial respiration.

We have demonstrated that >60 mitochondrial proteins are up-regulated in mammospheres derived from breast cancer cell lines (MCF7 and T47D), relative to cells grown in monolayers. Furthermore, pharmacological inhibition with oligomycin A, an inhibitor of the ATP synthase, greatly reduced mammosphere formation [11]. More importantly, treatment with doxycycline, an FDA-approved antibiotic targeting mitochondrial ribosomes as a known ‘side effect’, inhibited the formation of tumor-spheres generated from cell lines across several different tumor types, as well as from primary tumor samples [12].

These studies indicate that mitochondria in TICs are potentially druggable targets for the prevention of tumor recurrence and metastasis. The aim of our current study was to further validate mitochondrial function as a critical target for the development of new anti-cancer therapies. Here, we employed XCT790 as an investigational compound to inhibit mitochondrial oxidative phosphorylation [15]. XCT790 is a well-established inverse agonist of Estrogen-Related Receptor α (ERRα), which functions as an essential cofactor of PGC1α. Importantly, PGC1α is required for the transcription of nuclear-encoded mitochondrial genes and drives mitochondrial biogenesis [16, 17]. XCT790 has been shown to inhibit colony formation in soft agar induced by PGC1α [18], and to induce cell death in chemo-resistant cancer cells [19]. However, to our knowledge, no studies have previously investigated the effects of XCT790 on the propagation and survival of TICs.

Here, our results show that XCT790 inhibits the proliferation of TICs from MCF7 breast cancer cells, as assessed using i) mammosphere formation and by ii) CD44/CD24 immuno-staining, coupled with FACS analysis. We also demonstrate that XCT790 suppresses the activation of well-established pathways governing TIC proliferation and survival, including Sonic hedgehog, TGFβ-SMAD, STAT3, and Wnt signaling. As expected, XCT790 profoundly reduced mitochondrial respiration. Importantly, XCT790-induced TIC inhibition could be rescued by treatment with Acetyl-L-Carnitine (ALCAR), a mitochondrial fuel and cofactor, which enhances mitochondrial oxidative metabolism and biogenesis [20]. We also show that overexpression of ERRα is sufficient to augment MCF7 cell mammosphere formation, which can be blocked by treatment with mitochondrial inhibitors, such as XCT790 and oligomycin A, an inhibitor of ATP synthase. Finally, we show that mammosphere formation driven by FOXM1, a transcription factor downstream of Wnt/β-catening signaling that governs stemness [21-24], can also be blocked by treatment with three different classes of mitochondrial inhibitors (XCT790, oligomycin A, or doxycycline).

Thus, our results firmly establish that mitochondrial biogenesis and metabolism are required for the survival of TICs, indicating that mitochondrial-targeted therapies are a promising novel strategy for targeting TICs for the treatment of relapsed and resistant cancers. Mitochondrial-targeted therapies should also be considered for cancer prevention.

## RESULTS

### Mammosphere formation in MCF7 cells depends on mitochondrial function

We have previously shown that mitochondrial proteins are up-regulated in mammospheres derived from breast cancer cell lines (MCF7 and T47D), relative to cells grown in monolayers [11]. Furthermore, pharmacological inhibition with oligomycin A, an inhibitor of ATP synthase, greatly reduced mammosphere formation [11]. More importantly, treatment with doxycycline, an FDA-approved antibiotic that targets mitochondrial ribosomes as a known ‘side effect’, inhibited the formation of tumor-spheres derived from 12 cell lines across eight different tumor types, as well as from primary tumor samples [12].

**Figure 1:**
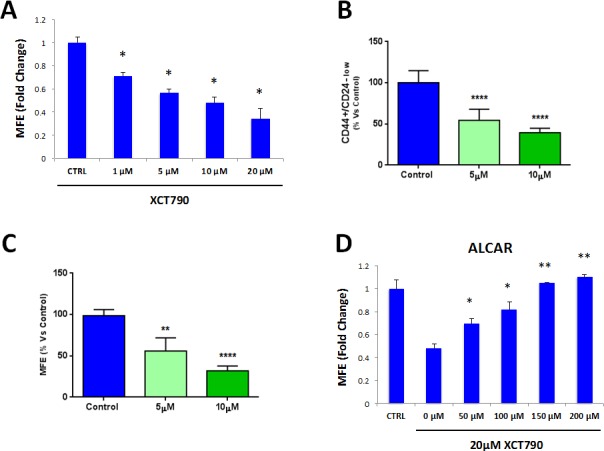
MCF7 cell 3D-spheroid formation depends on mitochondrial function **A.** The well-established ERRα inverse agonist, namely XCT790, dose-dependently inhibits MCF7 mammosphere formation, with an IC-50 of 10 μM. ERRα is a cofactor of PGC1α, which is required for the transcription of mitochondrial genes and mitochondrial biogenesis. *p < 0.01 evaluated by Student's t test. **B.** MCF7 cells were pre-treated with XCT790 (at 5 or 10 μM) as monolayers for 2 days and then re-plated on low-attachment plates in the absence of XCT790, for anoikis assay for 10 hours. Expression of TIC markers CD24 and CD44 was analyzed by FACS. Note that XCT790 pre-treatment dose-dependently reduced the number of CD44(+)high/CD24(−)low cells, which are considered the TICs population. ****p < 0.00001 evaluated with one-way ANOVA. **C.** MCF7 cells were pre-treated with XCT790 (at 5 or 10 μM) as monolayers for 2 days and then re-plated on low-attachment plates in the absence of XCT790, for mammosphere assay for 5 days. Under these conditions, XCT790 pre-treatment dose-dependently reduced MCF7 cell mammosphere formation, by up to ~70%. **p < 0.001 and ****p < 0.00001 evaluated with one-way ANOVA. **D.** Treatment with the mitochondrial cofactor Acetyl L-Carnitine (ALCAR) rescues the decreased mammosphere formation induced by XCT790. Mammosphere formation was assessed upon treatment with 20 μM XCT790 and increasing concentration of ALCAR. Thus, mitochondrial function is required for the efficient clonal expansion and anchorage-independent growth of TICs. *p < 0.05; **p < 0.001, relative to XCT790 only treated cells (0μM ALCAR) evaluated by Student's *t* test. MFE: mammosphere forming efficiency.

To further investigate the role of mitochondria in TICs, here we employed a well-established inverse agonist of ERRα, namely XCT790. ERRα is a cofactor of PGC1α/β, which is required for the transcription of nuclear mitochondrial genes and mitochondrial biogenesis [16, 17]. As such, XCT790 is believed to suppress mitochondrial function. Notably, treatment with XCT790 dose-dependently inhibits MCF7 mammosphere formation, with an IC-50 of 10 μM (Figure 1A). We also validated that XCT790 dose-dependently reduces the protein expression of PGC1α in MCF7 cell monolayers by immuno-blot analysis (data not shown), by up to 70-80%, over this concentration range.

These results were independently confirmed by evaluating the expression of *bona-fide* TIC markers CD44/CD24 by FACS. Under these conditions, CD44(+)high/CD24(−)low cells are considered to represent the TIC sub-population. XCT790 treatment significantly reduced the number of CD44(+)high/CD24(−)low cells in a dose-dependent fashion, relative to vehicle alone controls (Figure 1B).

We next set out to investigate if XCT790 can target TICs in the presence of the total cancer cell population. To this end, MCF7 cells were treated with XCT790 (at 5 or 10 μM) as monolayers for 2 days and then re-plated on low-attachment plates in the absence of XCT790, to generate mammospheres for 5 days. Under these conditions, XCT790 pre-treatment dose-dependently reduced MCF7 cell mammosphere formation, by up to ~70% (Figure 1C), indicating that XCT790 can target the TIC population also when present in a heterogeneous cell population.

Also, we asked if decreased mammosphere formation induced by XCT790 could be rescued by treatment with the mitochondrial cofactor Acetyl-L-Carnitine (ALCAR). ALCAR plays a key role in mitochondrial oxidative metabolism, by enhancing fatty acid β-oxidation [20]. ALCAR stimulates mitochondrial biogenesis and is also directly converted to acetyl-CoA, a mitochondrial fuel [20]. To this end, mammosphere formation was assessed after treatment with XCT790 (at 20 μM) and increasing concentrations of ALCAR. Figure 1D shows that ALCAR rescues the decrease in mammosphere formation induced by XCT790, in a dose-dependent manner. Thus, mitochondrial function is required for the efficient clonal expansion and anchorage-independent growth of TICs.

**Figure 2:**
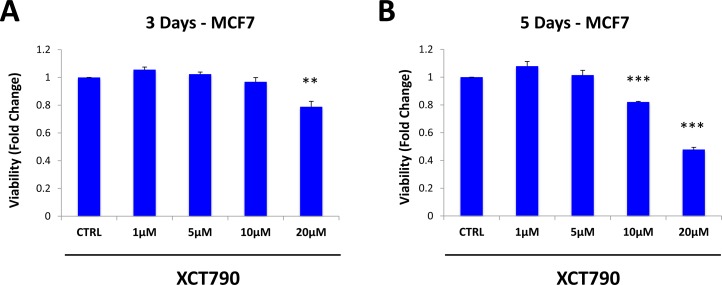
XCT790 preferentially reduces the viability of MCF7 cells in mammospheres, relative to bulk cancer cells MCF7 cells were treated with XCT790 (1, 5, 10, 20 μM) as monolayers for 3 days **A.** or 5 days **B.** Cell viability was assessed by SRB assay. Note that 5-day treatment did not affect the viability of the MCF7 cell monolayers as profoundly as MCF7 cell mammospheres. For example, treatment with 10 μM XCT790 reduces mammosphere formation by 50% (Figure 1A), whereas viability of monolayer cells is reduced by 20%. **p < 0.01; ***p < 0.001 evaluated by Student's *t* test.

We then examined if XCT790 affects the viability of the total cancer cell population, or if it specifically inhibits the viability of MCF7 cells in mammospheres. To this end, MCF7 cells were treated with increasing concentrations of XCT790 as monolayers for 3 days (Figure 2A) or 5 days (Figure 2B). Cell viability was then assessed using the SRB assay. Note that 5-day treatment did not affect the viability of the MCF7 cell monolayers, as profoundly as MCF7 cell mammospheres (Figure 2B). For example, treatment with 10 μM XCT790 reduces mammosphere formation by 50% (Figure 1A), whereas the viability of monolayer cells is reduced by only 20%. Thus, XCT790 preferentially reduces the viability of MCF7 cell mammospheres, relative to bulk cancer cells.

### XCT790 inhibits the activation of several stem cell related signaling pathways

**Figure 3:**
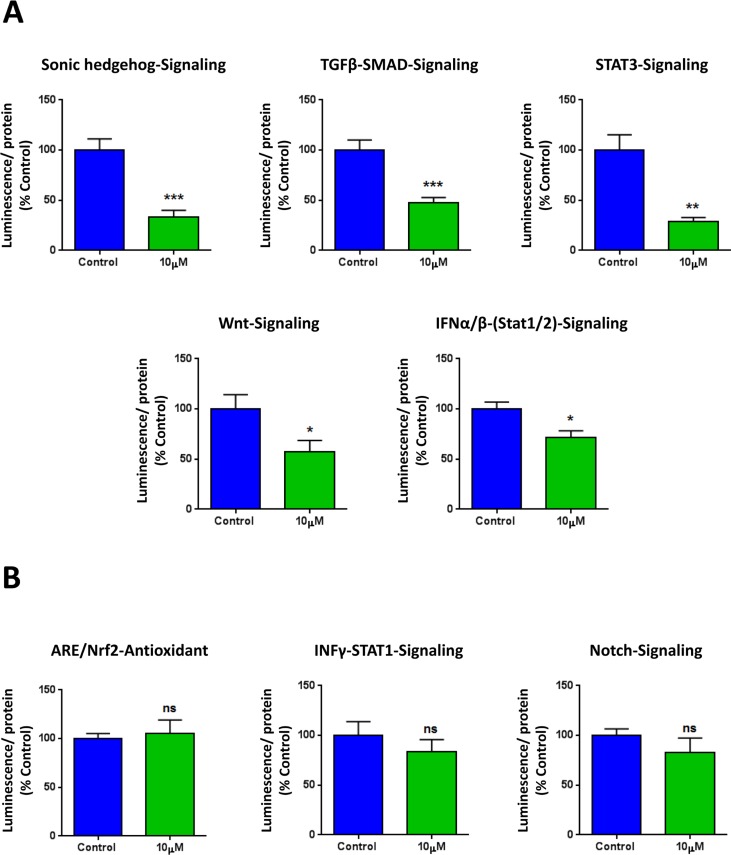
XCT790 inhibits signaling pathways related to cancer stem cells, and interferon MCF7 breast cancer cells carrying luciferase-reporters (Cignal, QIAGEN) were generated to monitor the activation of a variety of signaling networks, including Sonic hedgehog TGFβ-SMAD, STAT3, Wnt, Interferon (IFN)-α/β-STAT1/2, NRF2-dependent antioxidant responses, IFN-γ-STAT1 and Notch pathways. MCF7-Luc reporter cells were treated with XCT790 for 48 hours and luminescence was determined as a measure of pathway activation status. Luminescence was normalized by protein content. **A.** Note that XCT790 inhibits cancer stem cell signaling (Sonic hedgehog TGFβ-SMAD, STAT3, Wnt), as well as IFN-α/β-STAT1/2 signaling. **B.** No effects were observed for the NRF2-antioxidant responses, IFN-γ-STAT1 and Notch pathways. *p < 0.01; **p < 0.001; ***p < 0.0001, using Student's t test.

To further corroborate the idea that XCT790 inhibits cancer stem cell-like features, we next analyzed the effects of XCT790 on a series of well-established signaling pathways, which have been shown to promote stemness. For this purpose, we employed a panel of eight MCF7 cell lines carrying different luciferase reporters [25], to monitor the activation state of a variety of different signaling networks, including Sonic hedgehog, TGFβ-SMAD, STAT3, Wnt, Interferon (IFN)-α/β-STAT1/2, NRF2-dependent antioxidant responses, IFN-γ-STAT1 and Notch pathways. Notably, several pathways were significantly inhibited by XCT790 treatment, including stem cell signaling (Sonic hedgehog, TGFβ-SMAD, STAT3, Wnt) and IFN-α/β-STAT1/2 signaling (Figure 3A). However, no effects were observed on the NRF2-antioxidant response, IFN-γ-STAT1 and the Notch pathways (Figure 3B). Thus, XCT790 inhibits the activation of several signal transduction pathways related to cancer stem-like features.

### XCT790 inhibits mitochondrial respiration

**Figure 4:**
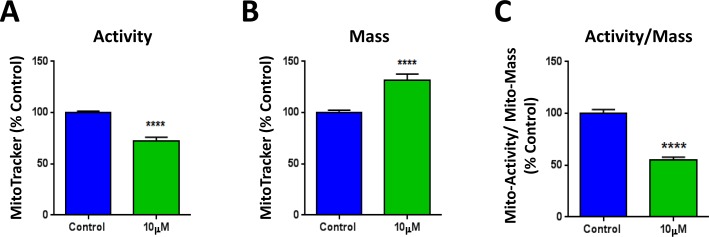
XCT790 induces a decrease in mitochondrial membrane potential, with an unexpected increase in mitochondrial mass MCF7 cells were treated with XCT790 (10 μM) as monolayers for 48 hours, stained with various MitoTracker probes and analyzed by FACS. **A.** XCT790 induces a decrease in mitochondrial membrane potential, as assessed with MitoTracker Orange, which accumulates in mitochondria with an active mitochondrial potential. **B.** XCT790 induces an unexpected increase in mitochondrial mass, as assessed with MitoTracker Green, which localizes to mitochondria regardless of mitochondrial membrane potential. **C.** Ratio of mitochondrial membrane potential (MitoTracker Orange), versus mitochondrial mass (MitoTracker Green) demonstrates that XCT790 induces a large decrease in mitochondrial membrane potential per mitochondria. ****p < 0.00001 evaluated with Student's *t* test.

Next, we set out to investigate the mechanism(s) by which XCT790 inhibits mammosphere formation and CSC features. XCT790 is an inverse agonist of ERRα, which is a cofactor of PGC1α required for the transcription of mitochondrial genes and mitochondrial biogenesis. Thus, the metabolic profile of MCF7 cells treated with XCT790 was analyzed. To this end, MCF7 cells were treated with XCT790 for 48 hours, stained with various MitoTracker probes and analyzed by FACS. Figure 4A shows that XCT790 induces a decrease in mitochondrial membrane potential, as assessed with MitoTracker Orange, which accumulates in mitochondria with an active mitochondrial potential. Surprisingly, XCT790 induces also an increase in mitochondrial mass, as assessed with MitoTracker Green (Figure 4B), localizing to mitochondria regardless of mitochondrial membrane potential. Similar results were obtained with MitoTracker Deep Red, which also measures mitochondria mass (data not shown). Analysis of the ratio of mitochondrial membrane potential versus mitochondrial mass indicates that XCT790 induces a profound decrease in mitochondrial membrane potential per mitochondria (Figure 4C).

**Figure 5:**
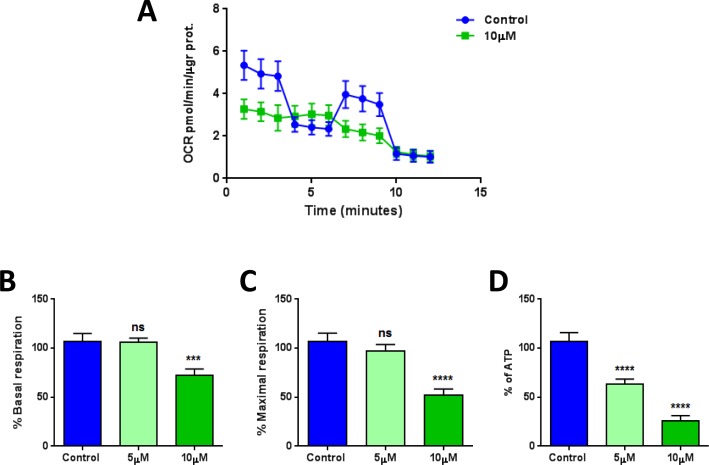
XCT790 quantitatively reduces mitochondrial respiration The metabolic profile of MCF7 cell monolayers treated with XCT790 (5 or 10 μM) for 2-days was examined using the Seahorse XFe96 analyzer. **A.** Oxygen consumption rate (OCR) is significantly reduced by treatment with 10 μM XCT790. **B.**, **C.**, **D.** Significant reductions in respiration (basal and maximal) and in ATP levels were observed upon XCT790 treatment. Thus, the rates of oxidative mitochondrial metabolism were significantly reduced by XCT790 treatment. ***p < 0.0001, ****p < 0.00001 evaluated with one-way ANOVA.

**Figure 6:**
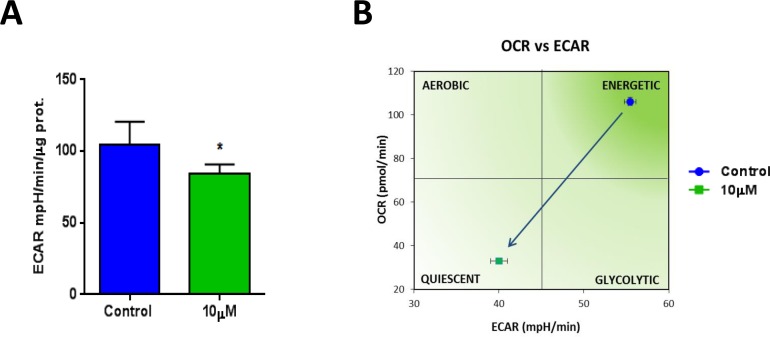
XCT790 shifts MCF7 cells from a highly energetic to a metabolically quiescent state **A.**Extracellular acidification rate (ECAR) of MCF7 cell monolayers treated with XCT790 (10 μM) for 2-days was assessed using the Seahorse XFe96 analyzer. Note that XCT790 significantly reduces ECAR, a marker of glycolysis. *p < 0.01 evaluated with Student's *t* test. **B.** OCR plotted against ECAR. Note that MCF7 cells treated with XCT790 were shifted towards a more metabolically quiescent state.

Also, the metabolic profile of MCF7 cells treated with XCT790 was examined using the Seahorse XFe96 analyzer, by employing a mitochondrial stress test. Notably, the oxygen consumption rate (OCR) was greatly reduced by treatment with XCT790 (Figure 5A). Further quantification revealed significant reductions in basal and maximal respiration (Figure 5B-5C), as well as ATP levels (Figure 5D), upon XCT790 treatment. Thus, XCT790 significantly reduces the rates of oxidative mitochondrial metabolism. To further validate these results, a glycolytic stress test was performed using the Seahorse XFe96 analyzer, by measuring the extracellular acidification rate (ECAR) of MCF7 cells treated with XCT790. Notably, XCT790 also significantly reduces ECAR, a marker of glycolysis (Figure 6A). Finally, plotting the ratio of OCR to ECAR demonstrates that XCT790 shifts MCF7 cells from a highly energetic to a metabolically quiescent state (Figure 6B).

### ERRα expression is both necessary and sufficient to increase mammosphere formation in MCF7 cells

**Figure 7:**
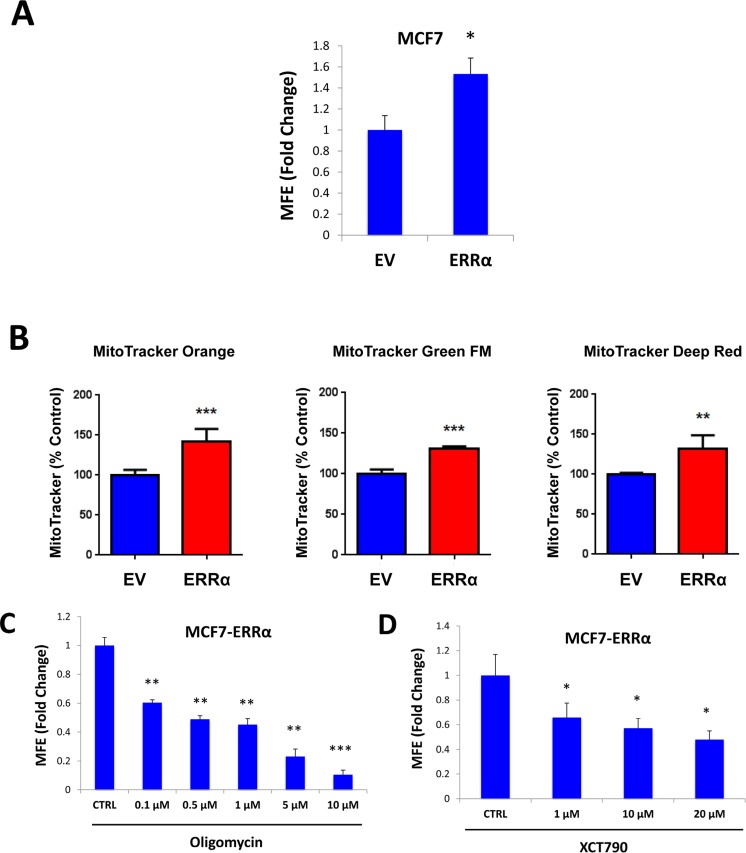
ERRα expression is required for 3D-spheroid formation in MCF7 cells ERRα was over-expressed in MCF7 cells using a lentiviral approach. Empty Vector (EV) cells were generated in parallel. **A.** MCF7 cells overexpressing ERRα show a 50% increase in mammosphere forming capacity, relative to EV controls. **B.** MCF7 cells over-expressing ERRα show an increase of mitochondrial membrane potential, as assessed by MitoTracker Orange staining, as well as an increase in mitochondrial mass, as assessed by MitoTracker Green and MitoTracker Deep Red staining, as expected. **C.** Oligomycin A, an inhibitor of mitochondrial ATP synthase, inhibits mammosphere formation in MCF7 cells overexpressing ERRα, indicating that mitochondrial function is required for ERRα-driven mammosphere formation. **D.** XCT790, an ERRα inverse agonist, inhibits mammosphere formation in MCF7 cells over-expressing ERRα. Thus, ERRα activity is required for the efficient clonal expansion of TICs. EV: Empty Vector. *p < 0.05, **p < 0.01, ***p < 0.001 evaluated by Student's *t* test.

XCT790 is an inverse agonist of ERRα. As XCT790 inhibits mammosphere formation, we then asked if ERRα expression is sufficient to promote 3D-spheroid formation in MCF7 cells. To this end, ERRα was over-expressed in MCF7 cells using a lentiviral approach. Empty Vector (EV) cells were generated in parallel. Figure 7A shows that MCF7 cells over-expressing ERRα show a 50% increase in mammosphere forming capacity. Notably, MCF7 cells over-expressing ERRα show increased mitochondrial membrane potential, as assessed by MitoTracker Orange, as well as increased mitochondrial mass, as assessed by MitoTracker Green and MitoTracker Deep Red, as expected (Figure 7B).

We then used a pharmacological approach to inhibit ERRα-driven mammosphere formation, using oligomycin A, a mitochondrial inhibitor, as well as XCT790, a specific ERRα inverse agonist. Importantly, mammosphere formation induced by ERRα expression was inhibited by either treatment with oligomycin A (Figure 7C), or XCT790 (Figure 7D). These results indicate that ERRα activity and mitochondrial function are normally required for the clonal expansion of TICs.

### Mammosphere formation driven by FOXM1 requires mitochondrial function

To examine if mitochondrial function is essential for TIC expansion driven by well-established stem-like signaling, we decided to overexpress FOXM1 in MCF7 cells. Empty Vector (EV) control cells were generated in parallel. The transcription factor FOXM1 was recently shown to induce the expansion of human normal stem cells, as well as of cancer stem-like cells [21-24]. Mechanistically, it is believed that FOXM1 acts downstream of key signaling pathways essential for stem cell regulation and tumorigenesis, such as Wnt/β-catenin and 14-3-3ζ signaling cells [22, 26].

**Figure 8:**
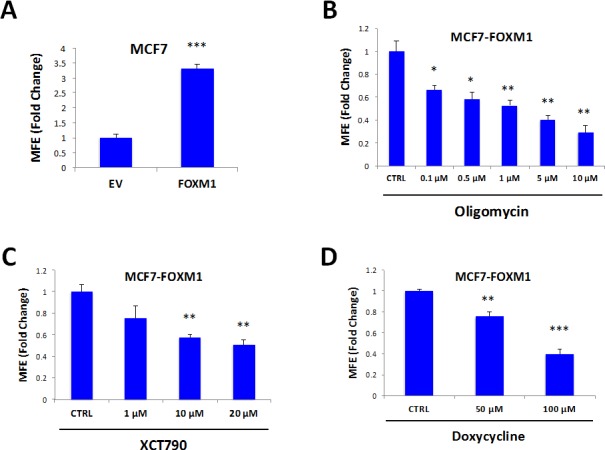
Mammosphere formation driven by FOXM1 requires mitochondrial function FOXM1 was over-expressed in MCF7 cells using a lentiviral approach. Empty Vector (EV) cells were generated in parallel. **A.** MCF7 cells over-expressing FOXM1 show a >3.3-fold increase in mammosphere formation. **B.** Treatment with oligomycin A, an inhibitor of the mitochondrial ATP synthase, inhibits mammosphere formation in MCF7 cells over-expressing FOXM1. **C.** Treatment with XCT790 inhibits mammosphere formation in MCF7 cells over-expressing FOXM1. **D.** Treatment with doxycycline, an FDA-approved antibiotic that we have recently shown to inhibit proliferation of TICs by targeting TIC mitochondria, inhibits mammosphere formation in MCF7 cells over-expressing FOXM1. Thus, mitochondrial function is required for FOXM1-driven mammosphere formation. EV: Empty Vector. *p < 0.05, **p < 0.01, ***p < 0.001 evaluated by Student's *t* test.

MCF7 cells over-expressing FOXM1 show a 3.3-fold increase in mammosphere formation, relative to empty-vector controls, as expected (Figure 8A). In order to better dissect the molecular changes driven by FOXM1, MCF7 cell monolayers over-expressing FOXM1 and EV control cells were subjected to proteomics analysis. Proteomic datasets of up-regulated proteins in the MCF7-FOXM1 cells were then analyzed with respect to their metabolic protein profiles.

Table 1 shows that several key molecules related to mitochondria, glycolysis and the EMT are up-regulated in FOXM1-over-expressing MCF7 cells, indicating that FOXM1-induced stemness is associated with increased metabolic flexibility. Notably, keratin-19 (KRT19), a well-established marker of circulating tumor cells and CSCs, is increased by >20-fold in MCF7-FOXM1 cells. Also, four key mitochondrial proteins (HSPD1, ACADVL, ATP5B, COX4I1) are increased by >10-fold each in MCF7-FOXM1 cells; SLC25A4 (the mitochondrial ATP/ADP exchanger) is increased by nearly 6-fold. Similarly, twenty-two other mitochondrial proteins are increased by >1.5 fold.

**Table 1:** MCF7-FOXM1 Cells Over-express Key Protein Molecules Functionally Related to Mitochondria, Glycolysis and the EMT

Symbol	Description	Fold-Upregulation	ANOVA
**Mitochondrial-related proteins (27)**			
HSPD1	HSP60, 60 kDa heat shock protein, mitochondrial	12.35	0.02
ACADVL	Acyl-Coenzyme A dehydrogenase, very long chain, mitochondrial	12.14	0.001
ATP5B	ATP synthase subunit beta, mitochondrial	10.74	0.0006
COX4I1	Cytochrome c oxidase subunit 4 isoform 1, mitochondrial	9.44	0.002
SLC25A4	Solute carrier family 25 (ADP/ATP translocase), member 4, mitochondrial	5.97	0.003
PRKDC	DNA-dependent protein kinase, catalytic (maintains mt-DNA copy number)	2.43	0.0005
ABAT	4-aminobutyrate aminotransferase, mitochondrial	2.28	0.0002
PMPCA	Mitochondrial-processing peptidase alpha subunit	2.21	0.03
CHCHD3	MICOS complex subunit MIC19, mitochondrial	2.04	0.03
NDUFS1	Mitochondrial NADH-ubiquinone oxidoreductase 75 kDa subunit	1.99	7.61E-05
HSPA9	Heat shock 70kDa protein 9 (Mortalin), mitochondrial	1.90	0.049
SUCLG2	Succinate-CoA ligase, GDP-forming, beta subunit	1.88	0.01
SLC25A3	Solute carrier family 25 (mitochondrial phosphate carrier), member 3	1.84	0.037
MTHFD2	methylene tetrahydrofolate dehydrogenase (NAD+dependent), mitochondrial	1.83	0.046
MDH2	Malate dehydrogenase 2, NAD (mitochondrial)	1.79	0.03
COX5A	Cytochrome c oxidase subunit 5A, mitochondrial	1.78	0.0045
TIMM9	Translocase of inner mitochondrial membrane 9	1.78	0.04
IDH2	Isocitrate dehydrogenase [NADP], mitochondrial	1.70	0.01
PDHA1	Pyruvate dehydrogenase E1 component subunit alpha, mitochondrial	1.67	0.037
MRPL49	39S ribosomal protein L49, mitochondrial	1.63	0.04
ATP5A1	ATP synthase subunit alpha, mitochondrial	1.60	0.03
HADH2	Hydroxyacyl-Coenzyme A dehydrogenase, type II, mitochondrial (HSD17B10)	1.55	0.04
HADHB	Trifunctional enzyme subunit beta, mitochondrial	1.55	0.049
ALDH18A1	Delta-1-pyrroline-5-carboxylate synthase, mitochondrial	1.55	0.02
GLRX5	Glutaredoxin-related protein 5, mitochondrial	1.97	0.03
PMPCB	Mitochondrial-processing peptidase subunit beta	1.69	0.03
MRPS22	28S ribosomal protein S22, mitochondrial	1.51	0.049
**Glycolysis and PPP (10)**			
G6PD	Glucose-6-phosphate 1-dehydrogenase	10.02	0.0003
GAPDH	Glyceraldehyde-3-phosphate dehydrogenase	8.35	0.003
ENO2	Gamma-enolase	7.72	6.65E-05
PKM1/2	Pyruvate kinase, muscle	5.49	0.0002
ALDOA	Fructose-bisphosphate aldolase	5.44	6.37E-05
ENO1	Alpha-enolase	2.81	0.02
PGD	6-phosphogluconate dehydrogenase, decarboxylating	1.86	0.005
TKT	Transketolase	1.81	0.004
TPI1	Triosephosphate isomerase 1	1.74	0.04
PGAM1	Phosphoglycerate mutase 1 (Brain)	1.62	0.0001
**EMT markers and Cytoskeletal proteins (31)**			
KRT19	Keratin, type I cytoskeletal 19	23.94	7.17E-06
PFN1	Profilin 1	10.85	0.046
ACTB	Actin, cytoplasmic 1	8.11	0.0002
ACTC1	Actin, alpha cardiac muscle 1	6.37	9.67E-05
ESPNL	Espin-like protein	5.74	2.35E-05
ACTBL2	Beta-actin-like protein 2	4.87	4.54E-06
TUBB2A	Tubulin beta-2A chain	4.70	0.0005
MYL6	Myosin light polypeptide 6	4.70	0.006
TUBB7	Tubulin beta-7 chain	4.40	0.003
EPPK1	Epiplakin	4.32	0.05
AGR2	Anterior gradient protein 2	3.84	0.0007
PAK2	Serine/threonine-protein kinase PAK 2	3.47	4.69E-05
PLEC	Plectin	3.12	0.001
CROCC	Rootletin	2.44	0.007
SPTBN1	Spectrin beta chain, non-erythrocytic 1	2.38	0.01
TPM2	Tropomyosin beta	2.35	0.002
TLN2	Talin-2	2.29	0.02
FLNB	Filamin-B	2.11	0.03
TUBB4	Tubulin beta-4 chain	2.05	0.03
DYNC1I2	Cytoplasmic dynein 1 intermediate chain 2	2.04	0.03
TMOD3	Tropomodulin-3	2.01	2.01E-05
FLNC	Filamin-C	1.93	0.008
ARPC2	Actin related protein 2/3 complex, subunit 2, 34kDa, isoform	1.84	0.047
APC	Adenomatosis polyposis coli	1.82	0.05
DSP	Desmoplakin	1.81	0.009
MYH14	Myosin-14	1.80	0.0006
TLN1	Talin-1	1.68	0.04
MYO1B	Myosin 1B	1.65	0.04
MSN	Moesin	1.59	0.02
TUBA1C	Tubulin alpha-1C	1.54	0.04
DYNLL2	Dynein light chain 2, cytoplasmic	1.52	0.048

Moreover, Table 2 shows that several key molecules related to protein synthesis are also up-regulated in FOXM1-overexpressing MCF7 cells. We have previously shown that protein synthesis is a mechanism for enhancing the proliferation of TICs, and that known inhibitors of protein synthesis, such as puromycin and rapamycin, are very effective at reducing mammosphere formation [27]. Similarly, the EMT is also regarded as a key feature of TICs [28, 29]. Consistent with the notion that FOXM1 drives stemness, ribosome-related proteins and EMT markers are increased by FOXM1 expression (Tables 1 and 2).

**Table 2:** MCF7-FOXM1 Cells Over-express Key Protein Molecules Functionally Related to Ribosomes and Protein Synthesis

Symbol	Description	Fold-Upregulation	ANOVA
**Ribosome-related proteins (9)**			
RPL15	60S ribosomal protein L15	5.49	0.003
RPL8	60S ribosomal protein L8	4.41	0.01
RPS3A	40S ribosomal protein S3A	1.78	0.046
RPL7	60S ribosomal protein L7	1.71	0.02
RPL10A	60S ribosomal protein L10A	1.69	0.006
RPL7A	60S ribosomal protein L7A	1.68	0.02
RRP1B	Ribosomal RNA processing protein 1 homolog B	1.66	0.05
MRPL49	39S ribosomal protein L49, mitochondrial	1.63	0.04
MRPS22	28S ribosomal protein S22, mitochondrial	1.51	0.049
**Translation initiation factors (4)**			
EIF4G3	Eukaryotic translation initiation factor 4 gamma 3	5.72	3.19E-06
EIF3C	Eukaryotic translation initiation factor 3 subunit C	2.44	0.001
EIF4B	Eukaryotic translation initiation factor 4B	1.83	0.04
EIF3S4	Eukaryotic translation initiation factor 3 subunit G	1.55	0.04
**Elongation factors (5)**			
EEF1A1	Elongation factor 1-alpha 1	5.63	0.0004
EEF1A2	Elongation factor 1-alpha 2	4.36	0.05
EEF1G	Elongation factor 1-gamma	3.03	0.003
EFTUD2	Elongation factor Tu GTP binding domain containing 2	2.84	4.94E-05
EEF1D	Elongation factor 1-delta	2.16	0.003
**Enzymes for tRNA synthesis (6)**			
GARS	Glycine--tRNA ligase	4.50	0.0006
DARS	Aspartate--tRNA ligase, cytoplasmic	2.28	0.01
TARS	Threonyl-tRNA synthetase, cytoplasmic	1.82	0.01
HARS	Histidine--tRNA ligase, cytoplasmic	1.82	0.03
RARS	Arginine--tRNA ligase, cytoplasmic	1.70	0.01
AARS	Alanine--tRNA ligase, cytoplasmic	1.56	0.03
**Protein folding chaperones (heat shock proteins) (8)**			
HSPD1	Heat shock 60kDa protein 1 (Chaperonin), mitochondrial	12.35	0.02
HSPB1	Heat shock protein beta-1	3.00	0.009
HSPA8	Heat shock cognate 71 kDa protein	2.20	0.002
HSP90AB1	Heat shock protein HSP 90-beta	1.97	9.13E-07
CLGN	Calmegin (ER chaperone)	1.59	0.026
HSPA1A	Heat shock 70 kDa protein 1	1.57	0.002
HSPH1	Heat shock 105kDa/110kDa protein 1	1.55	0.02
HSPA6	Heat shock 70 kDa protein 6	1.55	.006

To test if mitochondrial inhibitors could block FOXM1-driven mammosphere formation, we then used several independent pharmacological approaches. Interestingly, formation of FOXM1-driven mammospheres was inhibited by treatment with oligomycin A (Figure 8B), XCT790 (Figure 8C), and doxycycline (Figure 8D). Doxycycline is an FDA-approved antibiotic that we have recently shown to inhibit proliferation of TICs, by targeting their mitochondrial ribosomes [12]. Thus, mitochondrial protein translation and function are required for FOXM1-driven mammosphere formation.

### Mitochondrial function is required for 3D-spheroid formation using H295R cells

**Figure 9:**
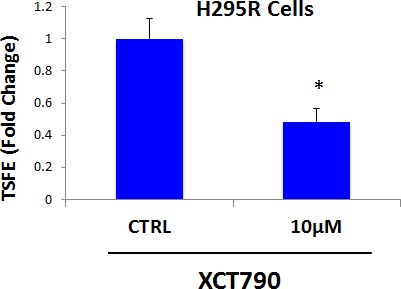
3D-spheroid formation in H295R cells is dependent upon mitochondrial function XCT790 dose-dependently inhibits 3D-spheroid formation in H295R cells, an adrenocortical carcinoma cell line, with an IC-50 of 10 μM. These results indicate that mitochondrial function is required for the 3D-spheroid formation in cell lines other than MCF7 breast cancer cells. TSFE, tumor-sphere forming efficiency; *p < 0.05 evaluated by Student's *t* test.

Finally, we asked if mitochondrial function is required for 3D-spheroid formation of other cell lines, such as H295R adrenocortical carcinoma cells. Figure 9 shows that XCT790 dose-dependently inhibits 3D-spheroid formation of the H295R adrenocortical carcinoma cell line, with an IC-50 of 10 μM. These results indicate that mitochondrial function is required for the 3D-spheroid formation of cell lines other than MCF7 breast cancer cells.

Quantitatively similar results were obtained when XCT790 was tested on several other epithelial cancer cell lines, indicating that the ability of XCT790 to inhibit 3D-spherioid formation reflects a general property of CSCs (data not shown).

### Relevance of FOXM1-related targets in human breast cancers

To assess the possible clinical relevance of our results, we also determined whether the proteomic targets that we identified in MCF7-FOXM1 cells were transcriptionally over-expressed in human breast cancer cells *in vivo*. Towards this end, we exploited a clinical data set of tumor samples from 28 breast cancer patients. These tumor samples were subjected to laser-capture micro-dissection, to separate epithelial cancer cells from adjacent tumor stroma [30].

Tables 3 and 4 present a summary of these findings. Overall, greater than fifty FOXM1 targets (related to mitochondria, glycolysis, the EMT, and protein synthesis) that we identified in MCF7-FOXM1 cells were also transcriptionally elevated in human breast cancer cells *in vivo*. As such, the new FOXM1 protein targets that we identified in MCF7-FOXM1 cells may be especially relevant for improving human breast cancer diagnosis and therapy.

**Table 3:** FOXM1 Targets are Transcriptionally Up-regulated in Human Breast Cancer: Mitochondria, Glycolysis and the EMT

Symbol	Gene Description	Up-regulation (fold-change)	P-value
**Mitochondrial-related proteins (23)**			
ATP5B	ATP synthase subunit beta, mitochondrial	5.04	2.75E-06
ATP5A1	ATP synthase subunit alpha, mitochondrial	5.01	3.09E-06
MRPL49	39S ribosomal protein L49, mitochondrial	4.94	3.93E-06
MDH2	Malate dehydrogenase 2, NAD (mitochondrial)	4.18	5.32E-05
GLRX5	Glutaredoxin-related protein 5, mitochondrial	4.07	7.83E-05
PMPCB	Mitochondrial-processing peptidase subunit beta	3.81	1.77E-04
SLC25A3	Solute carrier family 25 (mitochondrial phosphate carrier), member 3	3.76	2.09E-04
HSPA9	Heat shock 70kDa protein 9 (Mortalin), mitochondrial	3.69	2.64E-04
COX5A	Cytochrome c oxidase subunit 5A, mitochondrial	3.62	3.22E-04
TIMM9	Translocase of inner mitochondrial membrane 9	3.58	3.69E-04
HSPD1	HSP60, 60 kDa heat shock protein, mitochondrial	3.42	5.93E-04
COX4I1	Cytochrome c oxidase subunit 4 isoform 1, mitochondrial	3.39	6.61E-04
MRPS22	28S ribosomal protein S22, mitochondrial	3.27	9.31E-04
NDUFS1	Mitochondrial NADH-ubiquinone oxidoreductase 75 kDa subunit	3.20	1.15E-03
HADHB	Trifunctional enzyme subunit beta, mitochondrial	3.06	1.73E-03
SUCLG2	Succinate-CoA ligase, GDP-forming, beta subunit	3.03	1.89E-03
CHCHD3	MICOS complex subunit MIC19, mitochondrial	2.74	4.14E-03
MTHFD2	methylene tetrahydrofolate dehydrogenase (NAD+dependent), mitochondrial	2.67	4.98E-03
IDH2	Isocitrate dehydrogenase [NADP], mitochondrial	2.46	8.55E-03
PRKDC	DNA-dependent protein kinase, catalytic (maintains mt-DNA copy number)	2.14	1.85E-02
ABAT	4-aminobutyrate aminotransferase, mitochondrial	2.08	2.14E-02
PDHA1	Pyruvate dehydrogenase E1 component subunit alpha, mitochondrial	1.89	3.21E-02
**Glycolysis and PPP (7)**			
TPI1	Triosephosphate isomerase 1	4.21	4.88E-05
ALDOA	Fructose-bisphosphate aldolase	3.60	3.45E-04
PKM1/2	Pyruvate kinase, muscle	3.26	9.79E-04
GAPDH	Glyceraldehyde-3-phosphate dehydrogenase	2.97	2.22E-03
PGAM1	Phosphoglycerate mutase 1 (Brain)	2.55	6.87E-03
TKT	Transketolase	2.20	1.60E-02
ENO1	Alpha-enolase	1.96	2.75E-02
**EMT markers and Cytoskeletal proteins (11)**			
DSP	Desmoplakin	5.27	1.24E-06
FLNB	Filamin-B	4.81	6.21E-06
KRT19	Keratin, type I cytoskeletal 19	4.39	2.66E-05
DYNC1I2	Cytoplasmic dynein 1 intermediate chain 2	3.90	1.33E-04
MYL6	Myosin light polypeptide 6	3.74	2.22E-04
TUBA1C	Tubulin alpha-1C	3.30	8.64E-04
AGR2	Anterior gradient protein 2	2.94	2.44E-03
PAK2	Serine/threonine-protein kinase PAK 2	2.88	2.86E-03
TUBB2A	Tubulin beta-2A chain	2.63	5.56E-03
PFN1	Profilin 1	2.27	1.36E-02
ARPC2	Actin related protein 2/3 complex, subunit 2, 34kDa, isoform	2.04	2.33E-02

-Transcriptional profiling data derived from the analysis of N=28 breast cancer patients are shown, high-lighting the levels of fold-upregulation observed in the epithelial cancer cell compartment (relative to the tumor stroma), and corresponding p-values derived from the analysis of these clinical samples.

**Table 4:** FOXM1 Targets are Transcriptionally Up-regulated in Human Breast Cancer: Ribosomes and Protein Synthesis

Symbol	Gene Description	Up-regulation (fold-change)	P-value
**Ribosome-related proteins (9)**			
RPL7A	60S ribosomal protein L7A	5.23	1.41E-06
RPL7	60S ribosomal protein L7	5.21	1.53E-06
RPL10A	60S ribosomal protein L10A	5.09	2.35E-06
MRPL49	39S ribosomal protein L49, mitochondrial	4.94	3.93E-06
RPL15	60S ribosomal protein L15	4.60	1.28E-05
RPS3A	40S ribosomal protein S3A	4.59	1.35E-05
RPL8	60S ribosomal protein L8	3.86	1.51E-04
MRPS22	28S ribosomal protein S22, mitochondrial	3.27	9.31E-04
RRP1B	Ribosomal RNA processing protein 1 homolog B	2.21	1.58E-02
**Translation initiation factors (2)**			
EIF3C	Eukaryotic translation initiation factor 3 subunit C	4.48	1.94E-05
EIF4B	Eukaryotic translation initiation factor 4B	3.17	1.27E-03
**Elongation factors (3)**			
EEF1G	Elongation factor 1-gamma	3.71	2.44E-04
EEF1A1	Elongation factor 1-alpha 1	3.16	1.30E-03
EEF1D	Elongation factor 1-delta	2.50	7.67E-03
**Enzymes for tRNA synthesis (2)**			
DARS	Aspartate--tRNA ligase, cytoplasmic	3.43	5.87E-04
HARS	Histidine--tRNA ligase, cytoplasmic	2.42	9.55E-03
**Protein folding chaperones (heat shock proteins) (5)**			
HSP90AB1	Heat shock protein HSP 90-beta	4.93	5.93E-06
HSPD1	Heat shock 60kDa protein 1 (Chaperonin), mitochondrial	3.42	5.93E-04
HSPB1	Heat shock protein beta-1	3.27	9.51E-04
HSPH1	Heat shock 105kDa/110kDa protein 1	3.18	1.22E-03
HSPA8	Heat shock cognate 71 kDa protein	2.54	7.06E-03

-Transcriptional profiling data derived from the analysis of N=28 breast cancer patients are shown, high-lighting the levels of fold-upregulation observed in the epithelial cancer cell compartment (relative to the tumor stroma), and corresponding p-values derived from the analysis of these clinical samples.

## DISCUSSION

Here, we specifically tested the hypothesis that new mitochondrial biogenesis is required for the survival and propagation of stem-like cancer cells. For this purpose, we used an investigational inhibitor of the ERRα-PGC1α/β signaling pathway, namely XCT790, to interrogate the role of mitochondrial biogenesis and function in the anchorage-independent growth of CSCs. Importantly, treatment with XCT790 blocked mammosphere formation and prevented anoikis-resistance in CD44(+)high/CD24(−)low MCF7 cells. Mechanistically, XCT790 inhibited mitochondrial function (OCR) and effectively reduced signaling along a number of classical stem cell transduction pathways, such as the Hedgehog/GLI, TGFβ/SMAD, and Wnt/β-catenin signaling. Overexpression of either ERRα or FOXM1 (a down-stream target of Wnt-signaling [22]) in MCF7 cells significantly augmented mammosphere formation, which could be prevented by a number of mitochondrial inhibitors. In this regard, doxycycline treatment was sufficient to overcome the effects of FOXM1 on mammosphere formation. Interestingly, doxycycline is an FDA-approved antibiotic that has been used for nearly 50 years to treat a wide variety of bacterial and parasitic infections, without significant side effects. Finally, unbiased proteomics analysis of MCF7-FOXM1 cells revealed the up-regulation of specific mitochondrial proteins, glycolytic enzymes, EMT markers and components of the protein synthesis machinery. Importantly, these FOXM1 target proteins were also transcriptionally up-regulated in patient samples *in vivo*, in human breast cancer epithelial cells isolated by laser-capture micro-dissection, highlighting their clinical relevance.

Many other recent studies have also directly implicated augmented mitochondrial function in resistance to chemotherapy and radiation [31-36], in the survival of treatment-resistant cancer stem-like cells [37], in the propagation and motility of circulating tumor cells [38], as well as in tumor growth and cancer cell metastasis in pre-clinical animal models *in vivo* [39-44].

Consistent with our current findings, we have previously shown that a PGC1/NRF1 gene signature predicts tumor recurrence, metastasis and poor overall survival in ER(+)/Luminal-A breast cancer patients [45]. This PGC1/NRF1 gene signature was also elevated in >2,000 human tumors excised from breast cancer patients, including both ER(+) and ER(−) cases [45]. Moreover, recombinant over-expression of PGC1α/β, or other genes that promote mitochondrial biogenesis (MitoNEET/POLRMT), in MDA-MB-231 cells, was indeed sufficient to functionally increase tumor growth by up to ~3-fold [43].

Asymmetric cell division is an essential characteristic of stem cells. Interestingly, Sabatini, Weinberg and colleagues examined the segregation of “newly-synthesized” and “older” mitochondria in immortalized mammary epithelial cells, during asymmetric cell division, a property that is also a characteristic of CSCs [46]. They observed that “newly-synthesized” mitochondria were preferentially enriched in stem cells during asymmetric cell division, while “old” mitochondria were segregated into the daughter cells [46]. These results imply that asymmetric cell division in stem cells somehow requires new mitochondrial biogenesis, for the propagation and maintenance of the stem cell phenotype.

**Figure 10:**
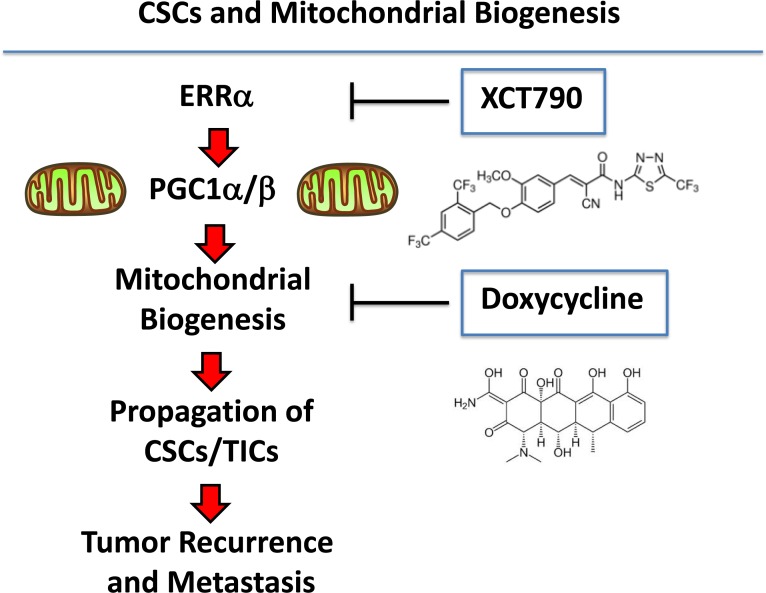
Understanding the role of mitochondrial biogenesis and function in the propagation of CSCs/TICs ERRα is a co-factor for PGC1α/β, which is a well-established transcription factor that is critical for driving new mitochondrial biogenesis. Here, we show that three different classes of mitochondrial inhibitors (XCT790, oligomycin A and doxycycline) all prevent the propagation of CSCs/TICs. XCT790 is an inverse agonist of ERRα. Oligomycin A is an inhibitor of the mitochondrial ATP-synthase (complex V) (not shown). Finally, doxycycline is an inhibitor of mitochondrial protein synthesis, as it binds directly to the small subunit of the mitochondrial ribosome. This reflects the fact that mitochondria were originally derived from aerobic bacteria that evolved over millions of years, to establish a symbiotic relationship with the host cell. The chemical structures of XCT790 and doxycycline are also shown.

As such, these findings may help to mechanistically explain our current results that new mitochondrial biogenesis is required for the efficient propagation of and survival of CSCs (Figure 10), as we have seen using the mammosphere and anoikis-resistance assays, to measure stem cell activity. Taken together, these data implicate mitochondrial biogenesis and mitochondrial function as critical targets for new drug discovery, to overcome tumor recurrence, distant metastasis and drug-resistance, especially in cancer patients with clinically advanced disease.

## MATERIALS AND METHODS

### Materials

MCF7 breast cancer cells were purchased from the ATCC. H295R adrenocortical carcinoma cells were a generous gift of Dr. Antonio Stigliano (University of Rome). Gibco-brand cell culture media (DMEM/F12) was purchased from Life Technologies. XCT790 was purchased from Tocris, oligomycin A and doxycycline were purchased from Sigma-Aldrich. Lentiviral vectors for the expression of ERRα (#Z1441) and FOXM1 (#U1376) were obtained commercially from Genecopoeia, along with the appropriate empty vector controls, in the Lv-105 (Puro^R^) vector system.

### Mammosphere culture

A single cell suspension was prepared using enzymatic (1x Trypsin-EDTA, Sigma Aldrich, #T3924), and manual disaggregation (25 gauge needle) to create a single cell suspension [8]. Cells were plated at a density of 500 cells/cm^2^ in mammosphere medium (DMEM-F12/B27/EGF(20ng/ml)/Pen-Strep) in non-adherent conditions, in culture dishes coated with (2-hydroxyethylmethacrylate) (poly-HEMA, Sigma, #P3932). Cells were grown for 5 days and maintained in a humidified incubator at 37°C at an atmospheric pressure in 5% (v/v) carbon dioxide/air. After 5 days for culture, spheres >50 μm were counted using an eye piece graticule, and the percentage of cells plated which formed spheres was calculated and is referred to as percentage mammosphere formation, and was normalized to one (1 = 100 %MSE, mammosphere forming efficiency). For pharmacological inhibition of mammosphere formation, cells were directly seeded on low-attachment plates in the presence of XCT790 or oligomycin or doxycycline at the indicated concentrations.

### Pre-treatment of monolayers with XCT790

After incubation with XCT790 (5 or 10 μM for 48 hours), MCF7 cell monolayers were trypsinized and seeded for mammosphere cultures for 5 days, without XCT790.

### Lentiviruses

Lentiviral plasmids, packaging cells and reagents were from Genecopoeia. 48 hours after seeding, 293Ta packaging cells were transfected with lentiviral vectors encoding ERRα, FOXM1 or empty vector (EX-NEG-Lv105), using Lenti-PacTM HIV Expression Packaging Kit according to the manufacturer's instructions. Two days post-transfection, lentivirus-containing culture medium was passed through a 0.45 μm filter and added to the target cells (MCF7 cells) in the presence of 5μg/ml Polybrene. Infected cells were selected with a concentration of 1.5 μg/ml of puromycin.

### CD44/CD24 analysis

Following XCT790 treatment for 48 hours, the TIC population was enriched by seeding on low-attachment plates. Under these conditions, the non-TIC population undergoes anoikis (a form of apoptosis induced by a lack of cell-substrate attachment) and TICs are believed to survive. The surviving TIC fraction was analyzed by FACS analysis. Briefly, 1 × 10^4^ MCF7 monolayer cells were treated with XCT790 (5μM and 10 μM) for 48h in 6-well plates. Then, cells were trypsinized and seeded in low-attachment plates in mammosphere media. After 10h, MCF7 cells were spun down and incubated with CD24 (IOTest CD24-PE, Beckman Coulter) and CD44 (APC mouse Anti-Human CD44, BD Pharmingen cat.559942) antibodies for 15 minutes on ice. Cells were rinsed twice and incubated with LIVE/DEAD dye (Fixable Dead Violet reactive dye; Invitrogen) for 10 minutes. Samples were then analyzed by FACS (Fortessa, BD Bioscence). Only the live population, as identified by the LIVE/DEAD dye staining, was analyzed for CD24/CD44 expression. Data were analyzed using FlowJo software.

### Viability assay

Cell viability was assessed by sulphorhodamine (SRB) assay, based on the measurement of cellular protein content. After treatment with XCT790 for 3 or 5 days in 96 well plates, cells were fixed with 10% trichloroacetic acid (TCA) for 1h in cold room, and dried overnight at room temperature. Then, cells were incubated with SRB for 15 min, washed twice with 1% acetic acid, and air dried for at least 1h. Finally, the protein-bound dye was dissolved in 10 mM Tris pH 8.8 solution and read using the plate reader at 540 nm.

### Evaluation of CSC signalling pathways

The Cignal Lenti reporter assay (luc) system (Qiagen) was chosen for monitoring the activity of several signaling pathways in MCF7 cells [25]. The responsive luciferase constructs encode the firefly luciferase reporter gene under the control of a minimal (m)CMV promoter and tandem repeats of response elements for each pathway. The following constructs were used: TCF/LEF(luc) for Wnt signaling (CLS-018L); STAT3(luc) for transcriptional activity of STAT3 (CLS-6028L); RBP-Jk(luc) for Notch-induced signaling (CLS-014L); ARE(luc) for Nrf2- and Nrf1-mediated antioxidant response (CLS-2020L); GAS(luc) for IFNγ-induced Stat1-signaling (CLS-009L); ISRE(luc) for (IFN)-α/β-STAT1/2 signaling (CLS-008L); SMAD(luc) for TGFβ-induced signaling (CLS-017L); GLI(luc) for Sonic hedgehog signaling (CCS-6030L). Briefly, 1 × 10^5^ MCF7 cells were seeded in 12-well plates. Once cells were attached, the viral particles were diluted 1:10 in complete culture media containing polybrene (sc-134220, Santa Cruz), and added to the cells. Puromycin treatment (#P9620, Sigma) was started 48 hours later in order to select stably infected cells.

### Luciferase assay

Luciferase Assay System (E1501, Promega) was performed in all luciferase reporter MCF7 cells treated with XCT790. Briefly, 6 × 10^3^ MCF7 cells were seeded in black-walled 96-well plates and then were treated with XCT790 (10 μg/ml). As control, vehicle-treated cells were run in parallel. Four replicates were used for each condition. After 48 hours of treatment, luciferase assay was performed according to the manufacturer's instructions. Light signal was acquired for 2 minutes in photons/second in the Xenogen VivoVision IVIS Lumina (Caliper Life Sciences), and the results were analysed using the Living Image 3.2 sofware (Caliper Life Sciences). Luminescence was normalized using total proteins, as assessed with the Bradford protein assay.

### Mitochondrial staining

To measure mitochondrial activity, cells were stained with MitoTracker Orange (#M7510, Invitrogen), whose accumulation in mitochondria is dependent upon membrane potential. To measure mitochondrial mass, cells were stained with MitoTracker Green (#M7514 Invitrogen), or MitoTracker Deep Red (#M22426, Invitrogen), both localizing to mitochondria regardless of mitochondrial membrane potential. Briefly, MCF7 cells were treated with XCT790 for 48 hours. Cells were then incubated with pre-warmed MitoTracker staining solution (diluted in PBS/CM to a final concentration of 10 nM) for 30-60 min at 37 °C. All subsequent steps were performed in the dark. Cells were washed in PBS, harvested, and re-suspended in 300 μL of PBS. Cells were then analyzed by flow cytometry. Data analysis was performed using FlowJo software.

### Seahorse XFe96 metabolic flux analysis

Extracellular acidification rates (ECAR) and real-time oxygen consumption rates (OCR) for MCF7 cells treated with XCT790 or vehicle alone control were determined using the Seahorse Extracellular Flux (XF96) analyzer (Seahorse Bioscience, MA, USA). MCF7 cells were maintained in DMEM supplemented with 10% FBS (fetal bovine serum), 2 mM GlutaMAX, and 1% Pen-Strep. 7,000 cells were seeded per well into XF96-well cell culture plates, and incubated overnight at 37°C in a 5% CO_2_ humidified atmosphere. After 24h, cells were treated with XCT790 (5μM or 10 μM) for 48h. After 48h of treatment, cells were washed in pre-warmed XF assay media (for OCR measurement, XF assay media was supplemented with 10mM glucose, 1mM Pyruvate, 2mM L-glutamine and adjusted at pH 7.4). Cells were then maintained in 175 μL/well of XF assay media at 37°C, in a non-CO_2_ incubator for 1h. During the cell incubation time, we loaded 25 μL each of 80mM glucose, 9μM oligomycin, 1M 2-deoxyglucose (for ECAR measurement) and 25 μL each of 10μM oligomycin, 9μM FCCP, 10μM rotenone, 10μM antimycin A (for OCR measurement), in XF assay media into the injection ports in the XFe-96 sensor cartridge. ECAR and OCR measurements were normalized by protein content. Data set was analyzed by XFe-96 software and GraphPad Prism software, using one-way ANOVA and Student's t-test calculations. All experiments were performed in quintuplicate, three times independently, such that each data point represents the average of 15 replicates.

### Label-free quantitative proteomics analysis

Cell lysates were prepared for trypsin digestion by sequential reduction of disulphide bonds with TCEP and alkylation with MMTS [47]. Then, the peptides were extracted and prepared for LC-MS/MS. All LC-MS/MS analyses were performed on an LTQ Orbitrap XL mass spectrometer (Thermo Scientific, San Jose, CA) coupled to an Ultimate 3000 RSLCnano system (Thermo Scientific, formerly Dionex, The Netherlands). Xcalibur raw data files acquired on the LTQ-Orbitrap XL were directly imported into Progenesis LCMS software (Waters Corp., Milford, MA, formerly Non-linear dynamics, Newcastle upon Tyne, UK) for peak detection and alignment. Data were analyzed using the Mascot search engine. Five replicates were analyzed for each sample type (*N* = 5). Statistical analyses were performed using ANOVA and only fold-changes in proteins with a p-value less than 0.05 were considered significant.

### Data mining

To firmly establish the clinical relevance of our results from the quantitative proteomics analysis of mammosheres, we re-analyzed the transcriptional profiles of epithelial breast cancer cells and adjacent tumor stromal cells that were physically separated by laser-capture microdissection (from *N* = 28 human breast cancer patients) [30].

## Abbreviations

CSCs: cancer stem-like cells
TICs: tumor-initiating stem-like cells
